# Supplementary material for: Ribosome profiling analysis of eEF3-depleted Saccharomyces cerevisiae
Source: Sci Rep. 2019 Feb 28;9:3037. doi: 10.1038/s41598-019-39403-y (PMC6395859; doi:10.1038/s41598-019-39403-y)
Supplement: Supplementary file 1 — Supplementary information [file 41598_2019_39403_MOESM1_ESM.docx]

**Supplementary Information**

**Ribosome profiling analysis of eEF3-depleted *Saccharomyces cerevisiae***

Villu Kasari^1,2†^, Tõnu Margus^1,2,†^, Gemma C. Atkinson^1,*^, Marcus J.O. Johansson^1,*^, Vasili Hauryliuk^1,2,3,*^

^1^Department of Molecular Biology, Umeå University, SE-901 87 Umeå, Sweden

^2^Laboratory for Molecular Infection Medicine Sweden (MIMS), Umeå University, SE-901 87 Umeå, Sweden

^3^ University of Tartu, Institute of Technology, 50411 Tartu, Estonia

^†^ these authors contributed equally

^*^ denotes the corresponding authors

**Contact details of corresponding authors:**

Gemma C. Atkinson: gemma.atkinson@umu.se, +46 706070315

Marcus J.O. Johansson: marcus.johansson@umu.se, +46 7856767

Vasili Hauryliuk: vasili.hauryliuk@umu.se, +46 907850807

**Supplementary Text**

*Conditional depletion of eEF3*

One possible approach to control the active concentration of eEF3 is to use a temperature-sensitive version of the protein (ts mutant). Since no temperature sensitive variants of eEF3 that retain wild type functionality at permissive temperatures have been selected to date despite several attempts^1,2^, we opted for post-transcriptional regulation of eEF3 levels.

In an attempt to establish a rapid-response system for controlling eEF3 levels, we constructed a strain deleted for *YEF3* that was rescued by a *YEF3* gene on a plasmid (see below). The translational efficiency of the plasmid-encoded *YEF3* mRNA was controlled via a tetracycline-binding aptamer (Tc-aptamer) in the 5' untranslated region (5'-UTR)^3^. Upon addition of the drug, the element acts as a roadblock for scanning 43S preinitiation complexes, thus inhibiting translation initiation. To more rapidly deplete eEF3, we also fused the *YEF3* open reading frame (ORF) to the sequence for a degron (Ub-X-e^K^) targeted by the N-end rule pathway^4,5^. We tested a set of constructs, varying plasmid copy number (low and high), number of Tc-aptamers (one or two), and the identity of the encoded N-terminal residue in the N-degron (methionine, arginine or glutamic acid). However, even in the case of the most responsive constructs, efficient growth inhibition was not observed until after 7-8 hours (Supplementary Fig. S8a-c). Moreover, western blotting showed that eEF3 levels were only slightly reduced after two hours of tetracycline treatment, highlighting the inefficiency of the system, and the need for an alternative strategy (Supplementary Fig. S8d). We therefore opted to control the steady-state level of the protein using the methionine-repressible strategy that is described in the Main text.

**Supplementary Materials and Methods:**

*Plasmid constructions*

A low copy *URA3* plasmid harboring the *YEF3* gene (pRS316-*YEF3*) was constructed by cloning a BamHI/SacI *YEF3* DNA fragment including ≈300 bp of upstream and downstream sequences into the corresponding sites of pRS316^6^. The *YEF3* DNA fragment was amplified from BY4742^7^ genomic DNA using primers V22 (TATCGAGCTCAAGACTCCGTTTAATCAC) and V23 (TACTGGATCCCTATGATCCGTCACCTAT). A low-copy *LEU2* plasmid carrying *YEF3* (pRS315-*YEF3*) was constructed by cloning a BamHI/SacI DNA fragment from pRS316-*YEF3* into the corresponding sites of pRS315^6^.

Low and high copy *LEU2* plasmids expressing mRNA encoding eEF3 protein fused with N-terminal degron (Ub-X-e^K^) as well as 5´-UTR post-transcriptional control element(s) (Tc-aptamer(s); tc1 or tc1-tc2) under a constitutive P*_TDH3_* promoter were constructed as follows. First, the vector backbone was amplified either from pRS315^6^ or pRS425^8^ using primers V36 (GCTGCACTGCAGACGGTTATCCACAGAATCAG) and V37 (AGTCGTGAGCTCGTTAACTGTGGGAATACTCAG). The PCR product was trimmed at the SacI and PstI sites introduced by linker sequences in the primers. Second, the P*_TDH3_*-tc1 fragment was amplified from p*TDH3*-tc1-3xHA^3^ (Euroscarf) using primers V24 (TATCGAGCTCCTGCCATTTCAAAGAATACG) and V29 (AAAGTCTTGACGAAAATCTGCATTTTTGGCCTAGGTGGTC). The PCR product was trimmed with XmaJI. Third, the P*_TDH3_*-tc1 fragment was ligated to XmaJI-digested ´tc1-Ub-Arg-e^K^-YEF3´, ´tc1-tc2-Ub-Met-e^K^-YEF3´, and ´tc1-tc2-Ub-Glu-e^K^-YEF3´ synthetic dsDNA gBlocks (Supplementary Table S1, all from IDT Integrated DNA Technologies) containing the first 41 bp of the *YEF3* ORF. The resultant P*_TDH3_*-tc1-Ub-Arg-e^K^-*YEF3*´*,* P*_TDH3_*-tc1-tc2-Ub-Met-e^K^-*YEF3*´ and P*_TDH3_*-tc1-tc2-Ub-Glu-eK-*YEF3*´ DNA fragments were PCR amplified using primers V24 (TATCGAGCTCCTGCCATTTCAAAGAATACG) and V28 (GTTCTTCTAGAACCTTAATG) and digested with SacI and XbaI. Fourth, a *YEF3* DNA fragment was generated by digesting pRS315-*YEF3* with XbaI and PstI. Finally, the pRS425-P*_TDH3_*-tc1-Ub-Arg-e^K^-*YEF3*, pRS315-P*_TDH3_*-tc1-tc2-Ub-Met-e^K^-*YEF3*, and pRS425-P*_TDH3_*-tc1-tc2-Ub-Glu-eK-*YEF3* plasmids were obtained by ligation of the three DNA fragments. Plasmids were validated by restriction analysis and DNA sequencing.

## *Construction of yeast strains with post-transcriptionally controlled eEF3 levels*

Using the PCR-mediated strategy^9^ we deleted copy of *YEF3* in a diploid strain formed between BY4728 and BY4742^7^. The *kanMX6* cassette was amplified from pFA6a-kanMX6^9^ using primers V9 (GTGTATTTTTCTAGTTCGAATCCATCGATAACATTAAAAGCGGATCCCCGGGTTAATTAA) and V10 (AAAATCTTAAAGAAAACTGTGAAAGCAGTGATCAAAAAGAGAATTCGAGCTCGTTTAAAC). The heterozygous *yef3*Δ/*YEF3* strain was transformed with pRS316-*YEF3*, sporulated, and a rescued *yef3*Δ strain (VKY20, MATa *his3 leu2Δ0 lys2Δ0 yef3*Δ::*kanMX6* /pRS316-*YEF3*) obtained from a tetrad. The VKY20 strain was transformed with pRS315-*YEF3,* pRS425-P*_TDH3_*-tc1-Ub-Arg-e^K^-*YEF3*, pRS315-P*_TDH3_*-tc1-tc2-Ub-Met-e^K^-*YEF3*, or pRS425-P*_TDH3_*-tc1-tc2-Ub-Glu-eK-*YEF3.* After purification by single cell streaks on SC-leu plates, cells that had lost the *URA3* plasmid were selected on SC-leu plates containing 5-fluoroorotic acid (5-FOA)^10^.

*Western blotting*

Exponentially growing cells at OD_600_≈0.5 were harvested by centrifugation at 1,500 x g for 5 min, washed, and resuspended in breaking buffer containing 50 mM Tris pH 7.5, 150 mM NaCl, 5% glycerol 1 mM DTT, cOmplete^TM^ protease inhibitor cocktail (EDTA-free, Roche). After addition of glass beads (0.5 mm diameter), cells were disrupted using a FastPrep-24 homogenizer (MP Biomedicals) at a speed setting of 4 m/sec for two times 20 sec, with 1 min on ice between steps. 5 µg of each total protein lysate was separated on 12% (for ribosomal proteins) or 8% SDS-PAGE and transferred to PVDF membrane (Immobilon P^SQ^, Merk Millipore). PBS-T (1x PBS, 0.05% Tween-20) with 5% w/v nonfat dry milk was used for overnight blocking at 4°C and 1% milk in PBS-T was used for 1 h antibody incubations. 5x 5 min washes with fresh changes of PBS-T was used between antibody incubations. Rabbit anti-eEF3 (a gift from Prof. Akira Kaji, University of Pennsylvania, USA), rabbit anti-eEF2 (ED7002, Kerafast), rabbit Peroxidase-anti-peroxidase to detect TAP tag (Sigma, Lot #103M4822), rabbit anti-Rps8 (gift from Giorgio Dieci, Univ. Parma, Italy), rabbit anti-Rpl10 (a gift from Prof. Bernard Trumpower, Dartmouth Medical School, USA), and mouse anti-Pgk1 (459250, Invitrogen) primary antibodies were used. Goat anti-rabbit IgG-HRP (AS09 602, Agrisera) or sheep anti-mouse IgG-HRP (NA931, Amersham) secondary antibodies and either Pierce ECL (32106, Thermo Scientific) or WesternBright^TM^ Quantum (K-12042-D10, Advansta) HRP substrates were used for chemiluminescent detection. Results were recorded and processed with LAS 4000 imaging system (Fujifilm) using standard (sensitivity/resolution) image-gathering settings.

*Analysis of the ribosome profiling data*

Ribo-Seq data processing pipeline: Most analyses were performed using custom software written in Python 3 available in GitHub at https://github.com/GCA-VH-lab/RiboSeqPy. Data is downloadable from https://www.ebi.ac.uk/arrayexpress/). That is the first step of the pipeline written in the Param.in file of RiboSeqPy. Steps 2-8 for Ribo-Seq data (which involve preprocessing, removing noncoding RNA and aligning to the genome up to uncorrected metagene plots) are collected under Pipeline_part_1.py. Corrected P-site assignment and codon relative fold difference tables are calculated by Pipeline_part_2.py (steps 9-12 in Param.in). Both parts of the pipeline are controlled by variables in the Param.in file. The quality of codon periodicity for individual read lengths was estimated using Rp-Bp^11^, run with default parameters and P-site offsets for each read length that were determined from metagene plots (Pipeline_part_1.py step 8). Depending on the replicate, 73 to 87% of reads mapped once to the genome and were used for final P-site assignment. Mapped reads were normalised as reads per million (RPM) using the number of reads that mapped only once.

RNA-Seq data processing: Noncoding RNA reads were removed using Bowtie2^12^ with default parameters. Alignment to the genome and transcriptome was done using HISAT2^13^.

Position specific normalisation of Ribo-Seq density by RNA-Seq – Ribosomal load_Position_: Normalisation was done using the script normalise_to_RNAseq.py which uses as input (i) P-site assignment data in the HDF5 binary data format (https://github.com/h5py/h5py) (Step 9 of pipeline); (ii) RNA-Seq data aligned to the genome in bam file format. The script outputs an HDF5 file with RNA-Seq normalized values. Position-based RNA-Seq coverage in RPMs was calculated using Bedtools^14^. Position specific normalised data were used for metagene plots and polarity score calculations.

Differential gene expression analysis: Ribo-Seq and RNA-Seq reads were processed as described above, with one important modification, i.e. alignment to genome using HISAT2^13^ allowed mapping both once and twice reads (-k 2). Gene coverage in RPKMs was calculated using Cufflinks^15^ with multi read correction (--multi-read-correction). Ribosomal load per ORF (Ribosomal load_ORF_) was calculated as the ratio between Ribo-Seq_RPKM_ and RNA-Seq_RPKM_.

GO enrichment analysis: molecular functions, biological processes, and/or cellular components enriched among differentially expressed genes were identified using YeastMine Gene Ontology (GO) enrichment tools^16^. GO enrichment results are provided as Supplementary Dataset S2.

Statistical testing: we used the following functions of the SciPy module *stats* (accessed on 2018-08-14)^17^: *zscore* (for calculation of Z-score), *normaltest* (to test normality of distributions), *mannwhitneyu* (nonparametric Mann-Whitney rank test), *wilcoxon* (nonparametric Wilcoxon signed-rank test).

Visualisation of Ribo-Seq data in IGV: P-site assignment data were stored in binary HDF5 format (<https://github.com/h5py/h5py)> which were converted to bedgraph using the script hdf2bedgraph.py. These were visualised in the Integrative Genomics Viewer (IGV)^18^.

Analysis of relative fold differences in ribosomal density for A-, P- and E-site positions: The analysis was based on that implemented by the Vazquez-Laslop and Mankin labs^19,20^. The relative fold difference in ribosome density (FD_P-site codon_ or just FD for simplicity) is calculated using Pipeline_part_2.py (steps 11-12). The data are filtered by minimal coverage (≥6 raw counts per codons) and the resultant master table containing FD, Z-score, A-, E- and P-site codon identity well as the amino acid sequence of the 14 aa-long peptide corresponding to positions -10 to +3 (+1 corresponds to A-site) is available as Supplementary Dataset S3. The table contains the set of codons (362,469) common for both replicates, log_2_(FD), Z-scores and sequence data of codons in E-, P-, and A-site as well as peptide sequence corresponding to codons from -10 to +3. We have extracted three sets of entries from the full dataset: i) P-site overrepresented (>2 Z-score for both replicates) ii) P-site underrepresented (< -2 Z-score for both replicates, i.e. the intersect between the two replicates) and iii) P-site invariant, used as background (-0.5 < Z-score < 0.5 for both replicates, i.e. the intersect between the two replicates). These entries were used to i) calculate A-, P- and E-site overrepresentation by calculating the ratio of frequencies of a given amino acid in foreground (FDP-site codon > 2 or FDP-site codon < -2) in relation to background (0.5 < FDP-site codon > -0.5) (Supplementary Table S2) and ii) identify the overrepresented motifs in positions -5 to +1 using pLogo^21^ (probability logo). pLogo is a web tool for visualizing and discovering sequence motifs based on the automated motif-extraction algorithm motif-x^22^. It computes the binomial probability of the observed frequency of a given residue in the given position in a motif in the foreground (in our case a set of peptide motifs selected by FD_P-site codon_ > 2 or FD_P-site codon_ < -2) in respect to background (in our case a set of peptide motifs selected by 0.5 < FD_P-site codon_ > -0.5) expressed as log_10_-odds.

**Supplementary Figures:**

**
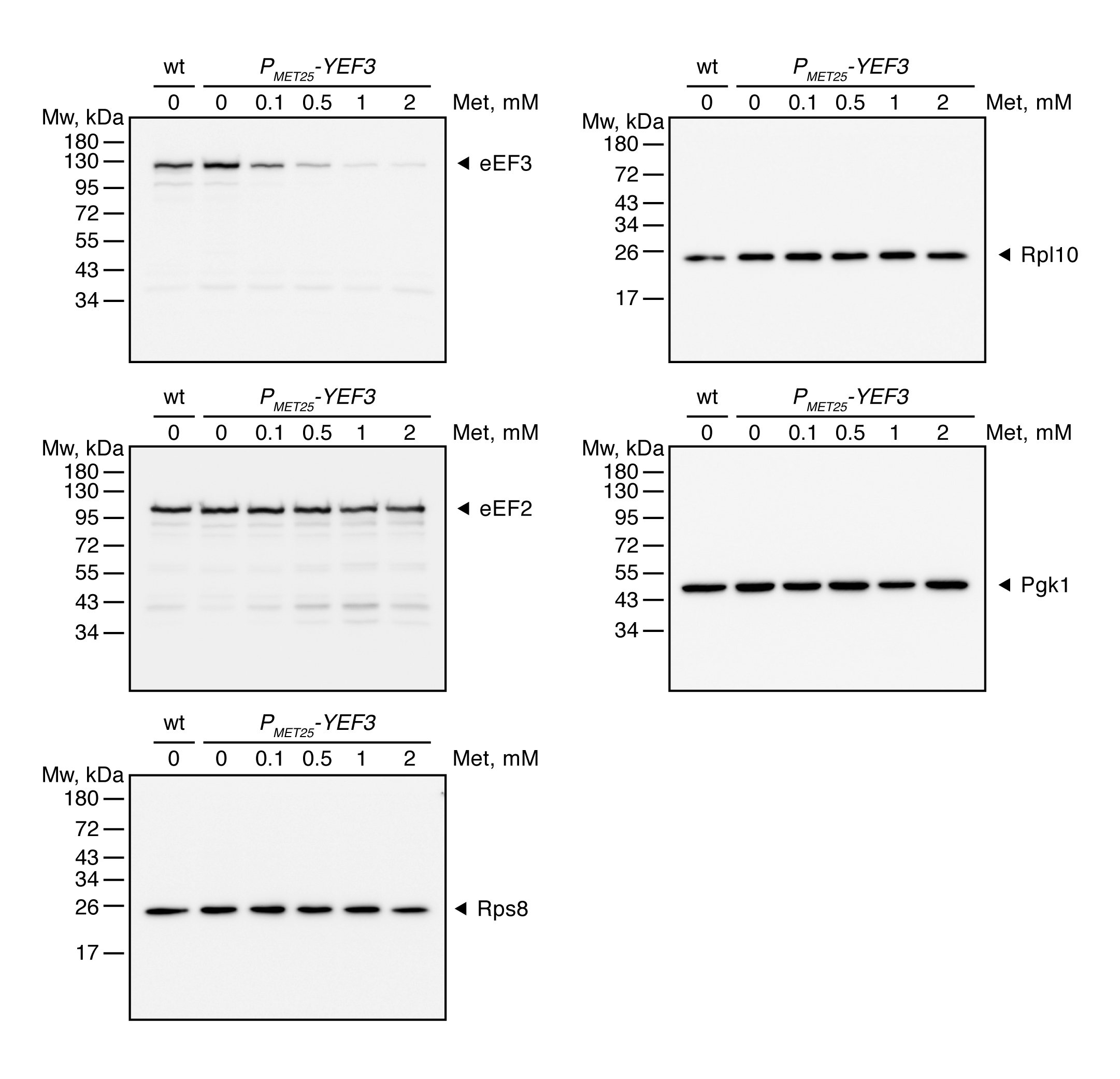
**

**Supplementary Fig. S1. Full-length images of western blots from Fig. 1c.**

**
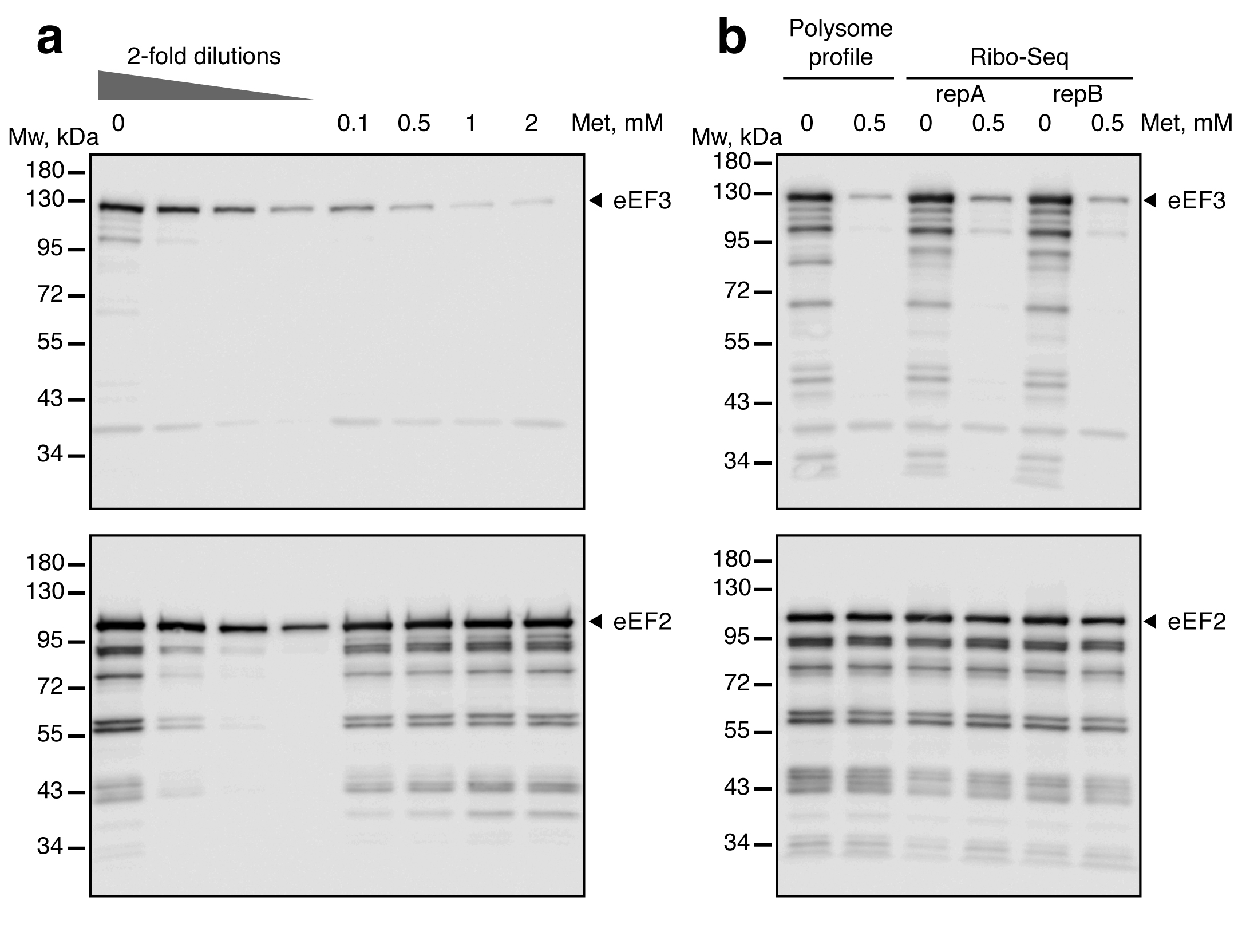
**

**Supplementary Fig. S2. Comparison of eEF3 levels in different cell extracts.** (**a**) Western blot analysis of the P*_MET25_-YEF3* (VKY8) strain grown in SC-met-cys medium supplemented with indicated methionine concentrations. Two-fold dilution series of the non-repressed *YEF3* extracts is shown to the left. (**b**) Western blot analysis of the cell extracts from the polysome profile and Ribo-Seq experiments. indicates the full-size protein band.

**­­
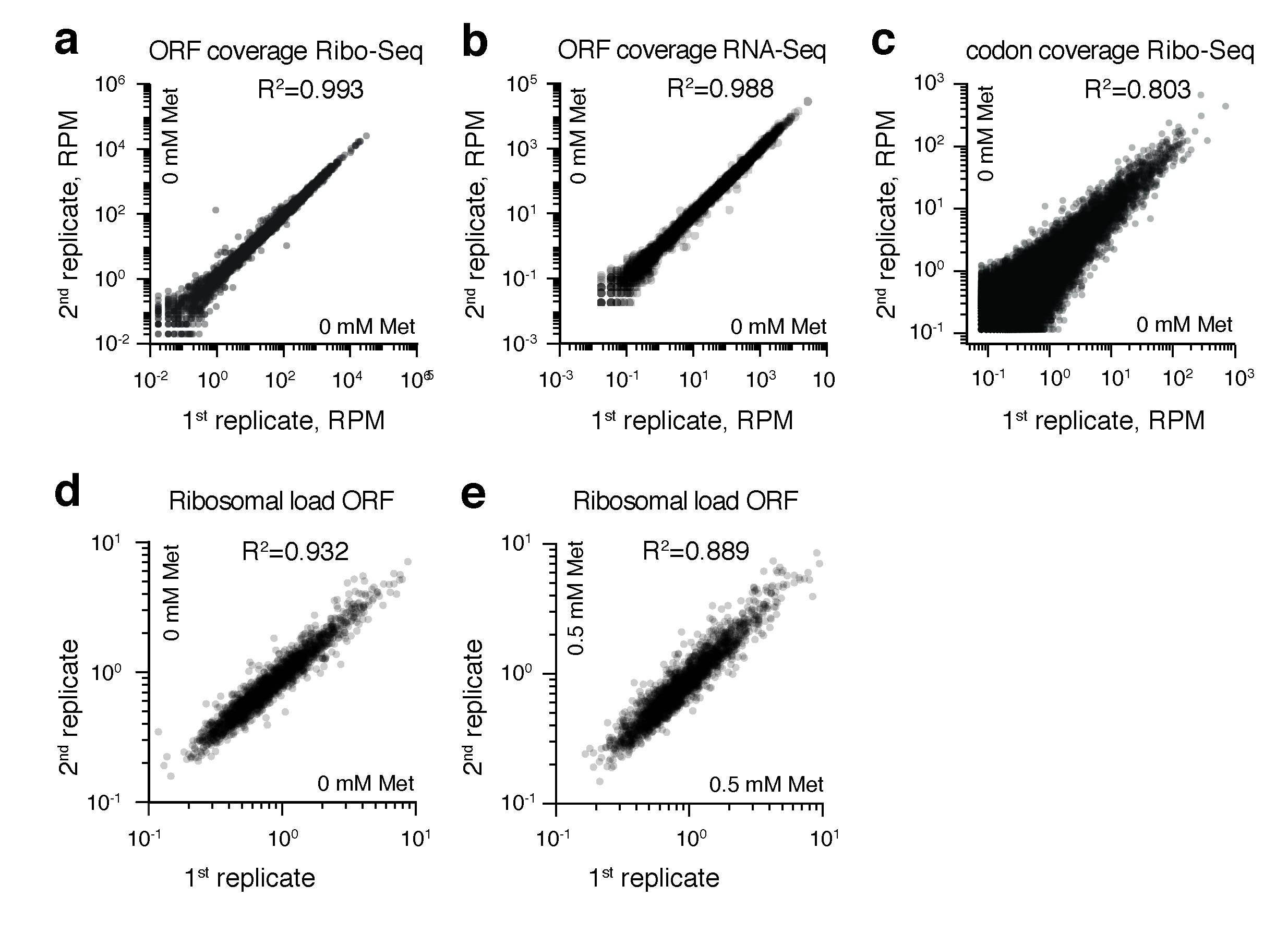
**

**Supplementary Fig. S3. Reproducibility of NGS libraries.** (**a**) RNA-Seq and (**b**) Ribo-Seq footprint densities in two biological replicates. Each data point (dot) corresponds to one ORF. (**c**) Ribosome footprint densities in two biological replicates. Each data point corresponds to one codon - all together there are approximately 360,000 codons in this comparison. (**d** and **e**) Ribosomal load per ORF for eEF3-proficient and eEF3-deficient conditions.

**
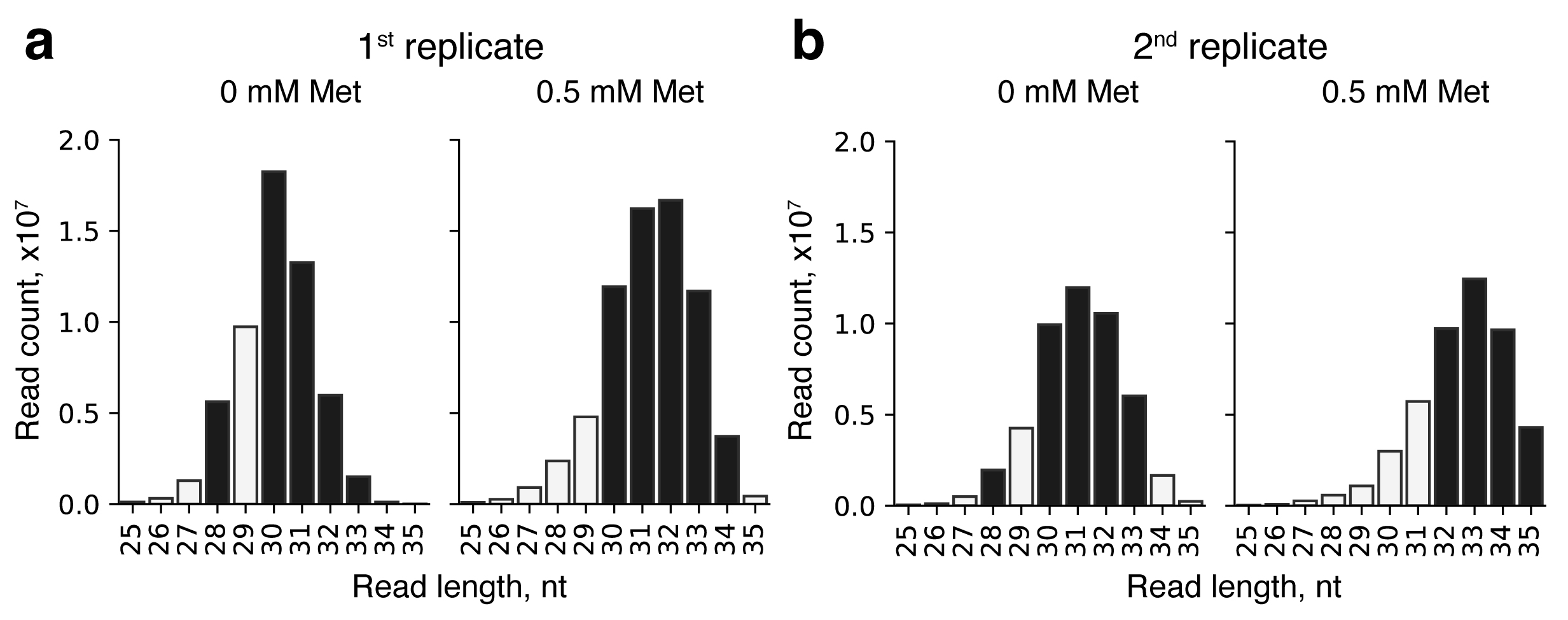
**

**Supplementary Fig. S4. Read length distribution of Ribo-Seq reads for two experimental replicates.** The quality of codon periodicity (good: black bars; poor: light gray) for each read length category was estimated with Rp-Bp^11^ with default settings.

**
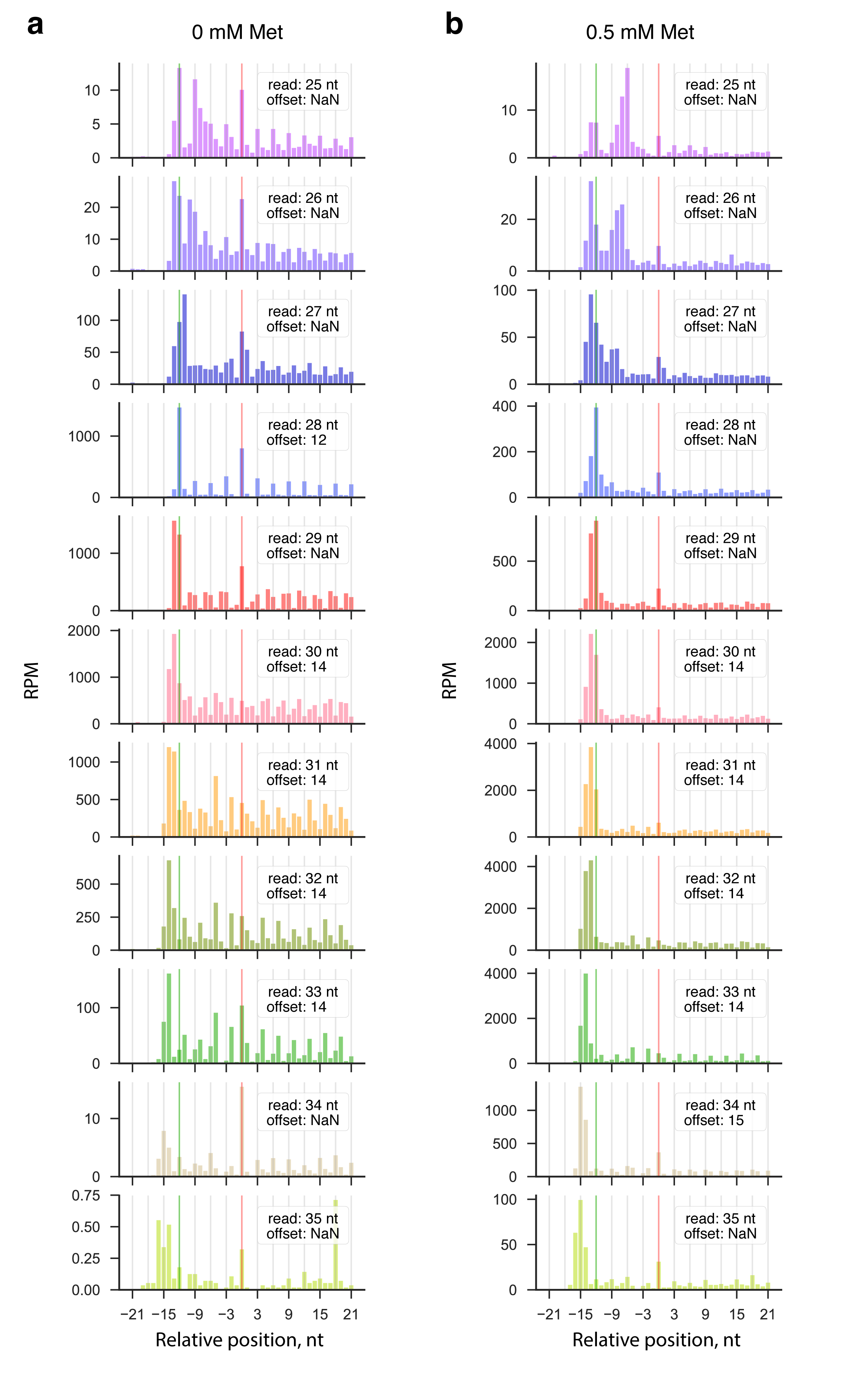
**

**Supplementary Fig. S5. Metagene analysis of Ribo-Seq data used to determine the read length-specific offsets for P-site assignment.** The data of 1^st^ biological replicate are plotted for eEF3-deficient (**a**) and eEF3-proficient (**b**) cells. The X-axis is the relative position around the 5' end of start codon (position 0). Negative values are upstream of the start codon. Vertical guide lines have a step of 3 nucleotides. Red and green guidelines correspond to 0 and -12 positions, respectively.

**
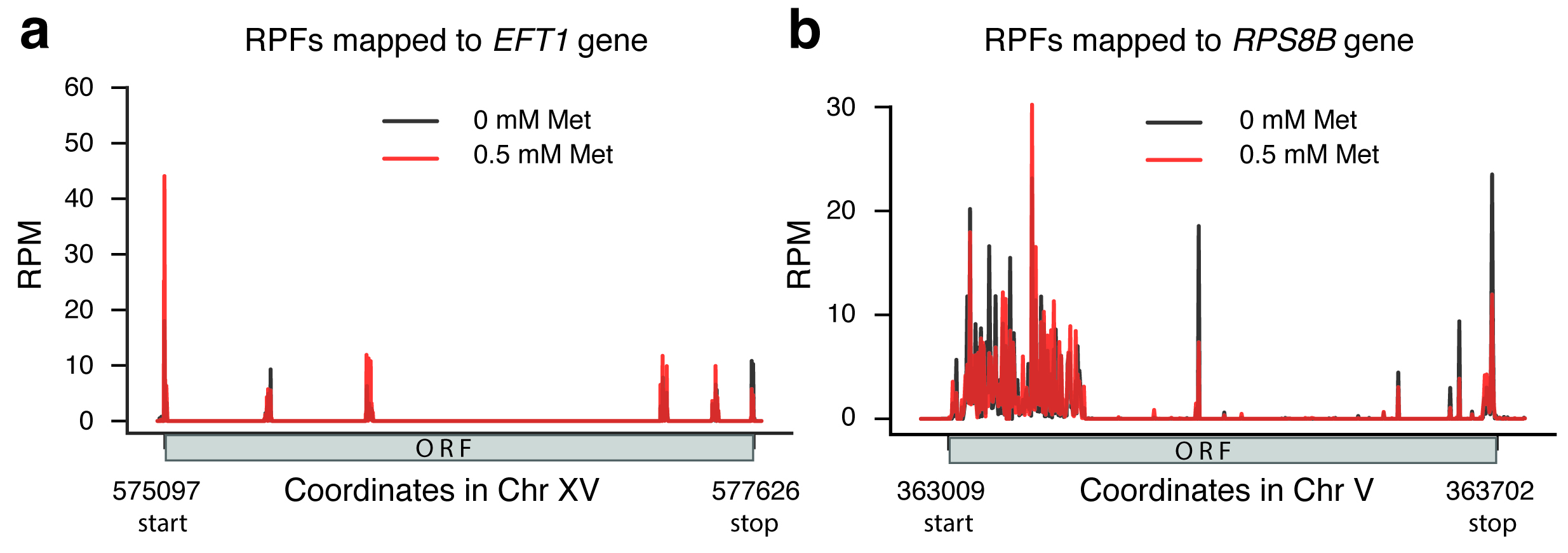
**

**Supplementary Fig. S6. RPF coverage of genes with highly similar paralogs.** *EFT1* (**a**) and *RPS8B* (**b**) gene coverage by Ribo-Seq reads mapped once; reads mapped twice and more are excluded. Coverage in eEF3-deficient (red) and eEF3-proficient (black) cells. The empty regions refer to stretches of identical sequences between paralogs *EFT1* and *EFT2* (**a**) as well as *RPS8A* and *RPS8B* (**b**).


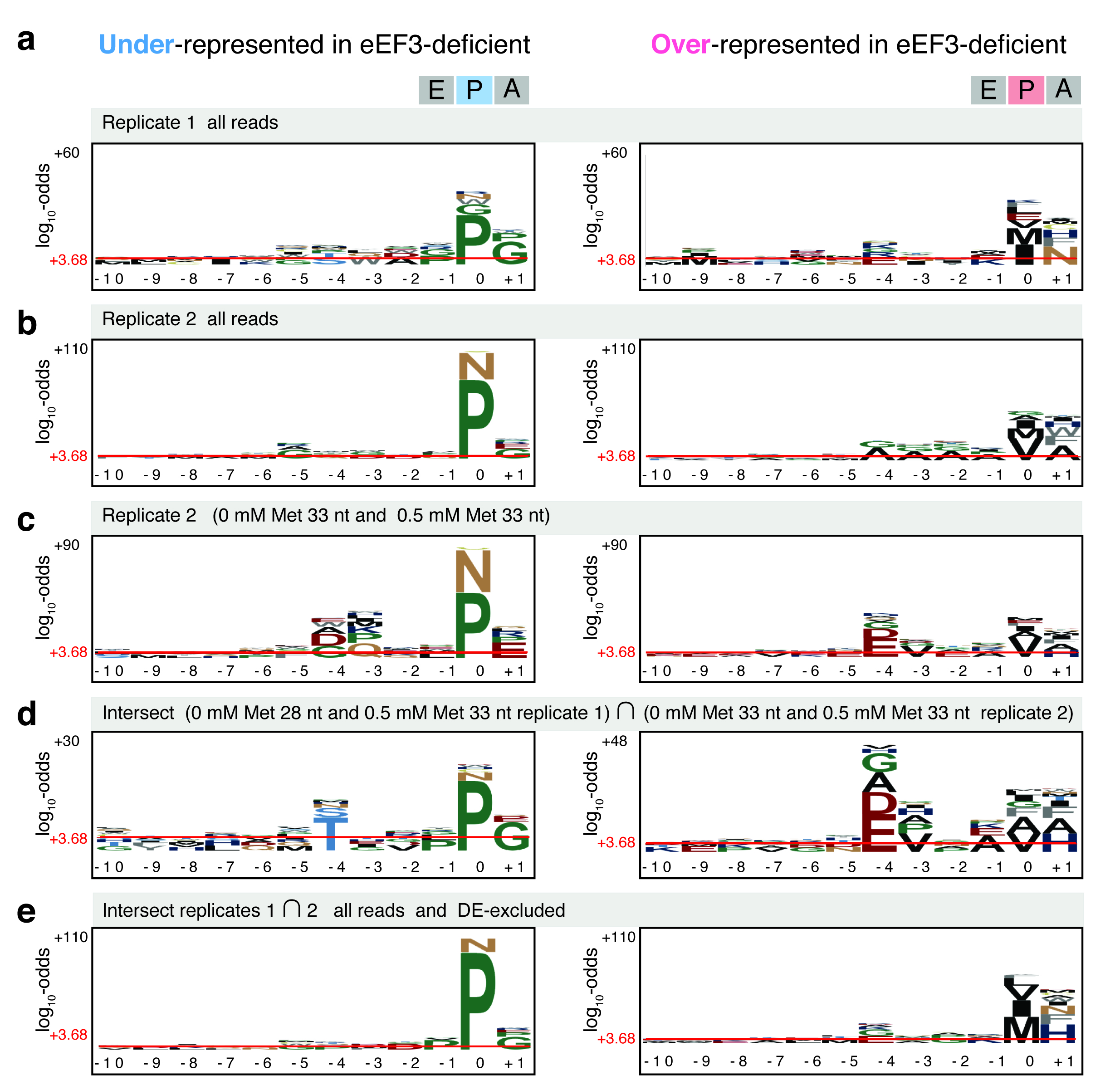


**Supplementary Fig. S7. Amino acid- and position-specific redistribution of ribosomal density upon depletion of eEF3 is robustly detected from Ribo-Seq data.** Analyses used either all of the read lengths that pass the quality check by Rp-Bp^11^ (**a**, **b** and **e**) or only specific read lengths that displayed good periodicity as per the metagene analysis (**c** and **d**). (**e**) Differentially expressed (DE) genes were selected on the basis of RNA-Seq and omitted from the dataset; otherwise the analysis was performed identically to that presented in the main text **Fig 5**. To achieve sufficient coverage (6 raw counts per codon), Z-score < -1.4 (143 codons) and > 1.5 (460 codons) was used for (**d**), Z-score < -2 and > 2 was used as the cut-off for (**e**), or 800 codons with highest or lowest FD were used for (**a**, **b** and **c**). Over- and underrepresentation of specific amino acids at positions relative to the P-site codon were calculated using pLogo^21^. Horizontal red lines represent the significance threshold (the log_10_-odds 3.68 and –3.68) corresponding to a Bonferroni corrected p-value of 0.05.

**
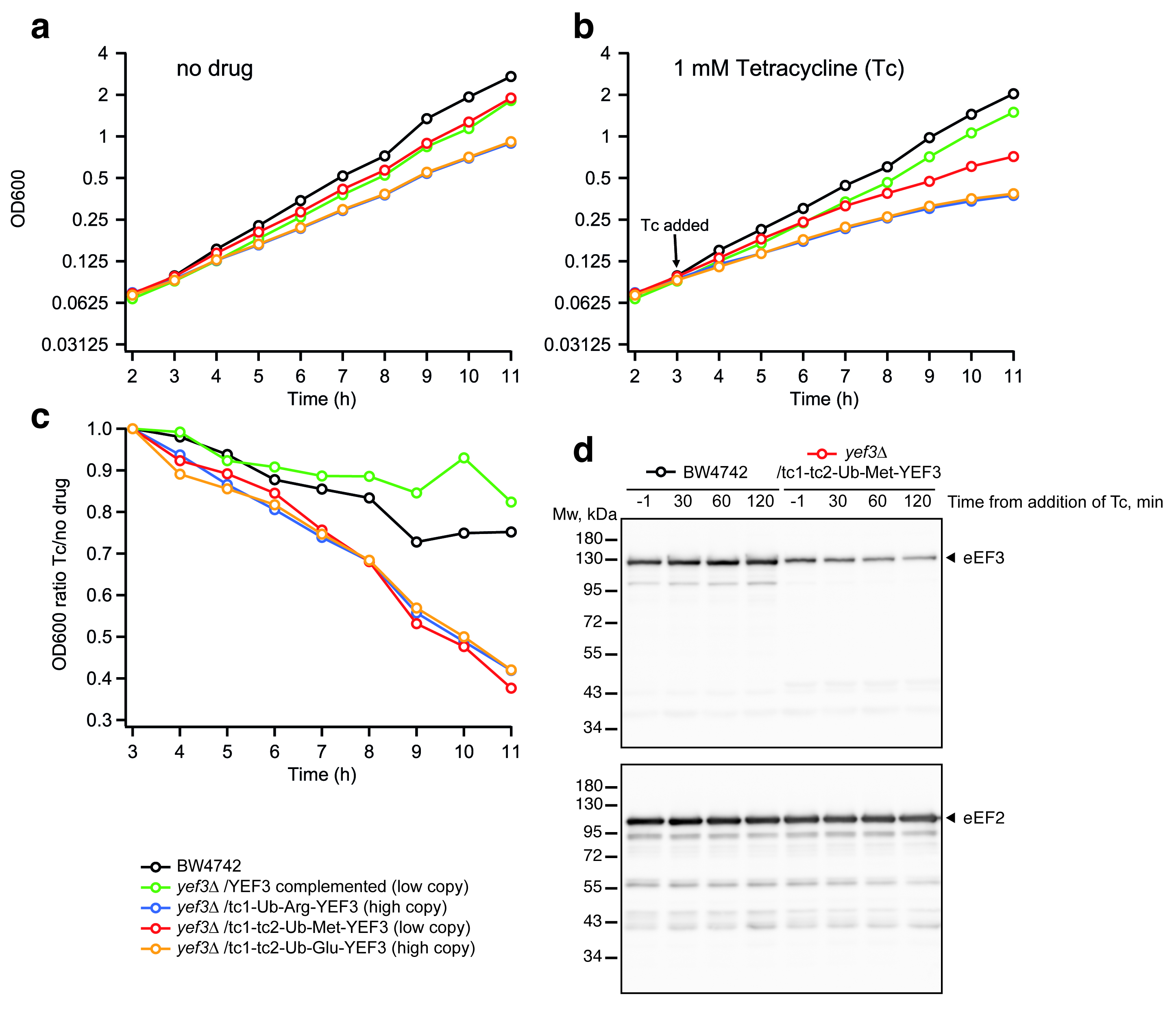
**

**Supplementary Fig. S8. Characterization of a genetic system for post-transcriptional regulation of eEF3 expression.** The wild-type (BY4742) and *yef3*Δ strain, complemented with the indicated plasmids, were grown overnight at 30°C in SC medium, diluted to OD_600_≈0.07 and grown for additional 3 hours. After that the cultures were split into two pre-warmed flasks, and one of the sub-cultures was supplemented with tetracycline (Tc) to a final concentration of 1 mM. OD_600_ was followed the absence (**a**) and presence (**b**) of the drug, and the ratio between the two OD_600_ values was calculated for each time point (**c**). Western blot analysis of cell extracts from wild-type and *yef3*Δ /pRS315-P*_TDH3_*-tc1-tc2-Ub-Met-e^K^-*YEF3* strains before and after addition of tetracycline (**d**).

**Supplementary Table S1: gBlocks used in this study.** XmaJI and XbaI restriction sites are underlined.

| **´tc1-Ub-Arg-e^K^-YEF3´ gBLOCK**  CGACCACCTAGGCCAAAAATGCAGATTTTCGTCAAGACTTTGACCGGTAAAACCATAACATTGGAAGTTGAATCTTCCGATACCATCGACAACGTTAAGTCGAAAATTCAAGACAAGGAAGGTATCCCTCCAGATCAACAAAGATTGATCTTTGCCGGTAAGCAGCTAGAAGACGGTAGAACGCTGTCTGATTACAACATTCAGAAGGAGTCCACCTTACATCTTGTGCTAAGGCTAAGAGGTGGTAGACACGGATCCGGAGCTTGGCTGTTGCCCGTCTCACTGGTGAAAAGAAAAACCACCCTGGCGCCCAATACGCAAACCGCCTCTCCCCGCGCGTTGGCCGATTCATTAATGCAGAGATCTATGTCTGATTCCCAGCAATCCATTAAGGTTCTAGAAGAACT |
| --- |
| **´tc1-tc2-Ub-Met-e^K^-YEF3´ gBLOCK**  CGTAGACCTAGGCCTTAAGAACCGGTAAAACATACCAGATCGCCACCCGCGCTTTAATCTGGAGAGGTGAAGAATACGACCACCTACCGGAAAAATGCAGATTTTCGTCAAGACTTTGACCGGTAAAACCATAACATTGGAAGTTGAATCTTCCGATACCATCGACAACGTTAAGTCGAAAATTCAAGACAAGGAAGGTATCCCTCCAGATCAACAAAGATTGATCTTTGCCGGTAAGCAGCTAGAAGACGGTAGAACGCTGTCTGATTACAACATTCAGAAGGAGTCCACCTTACATCTTGTGCTAAGGCTAAGAGGTGGTATGCACGGATCCGGAGCTTGGCTGTTGCCCGTCTCACTGGTGAAAAGAAAAACCACCCTGGCGCCCAATACGCAAACCGCCTCTCCCCGCGCGTTGGCCGATTCATTAATGCAGAGATCTATGTCTGATTCCCAGCAATCCATTAAGGTTCTAGAAGAACT |
| **´tc1-tc2-Ub-Glu-e^K^-YEF3´ gBLOCK**  CGTAGACCTAGGCCTTAAGAACCGGTAAAACATACCAGATCGCCACCCGCGCTTTAATCTGGAGAGGTGAAGAATACGACCACCTACCGGAAAAATGCAGATTTTCGTCAAGACTTTGACCGGTAAAACCATAACATTGGAAGTTGAATCTTCCGATACCATCGACAACGTTAAGTCGAAAATTCAAGACAAGGAAGGTATCCCTCCAGATCAACAAAGATTGATCTTTGCCGGTAAGCAGCTAGAAGACGGTAGAACGCTGTCTGATTACAACATTCAGAAGGAGTCCACCTTACATCTTGTGCTAAGGCTAAGAGGTGGTGAACACGGATCCGGAGCTTGGCTGTTGCCCGTCTCACTGGTGAAAAGAAAAACCACCCTGGCGCCCAATACGCAAACCGCCTCTCCCCGCGCGTTGGCCGATTCATTAATGCAGAGATCTATGTCTGATTCCCAGCAATCCATTAAGGTTCTAGAAGAACT |

**Supplementary Table S2. Overrepresentation of amino acids in E-, P-, A-sites upon eEF3 depletion.** Overrepresentation is calculated as the ratio of frequencies of a given amino acid in the foreground (FD_P-site codon_ > 2 or FD_P-site codon_ < -2; see Fig 5a) in relation to the background (0.5 < FD_P-site codon_ > -0.5; see Fig. 5a).

|  | FD_P-site codon_ > 2 | | | FD_P-site codon_ < -2 | | |  |
| --- | --- | --- | --- | --- | --- | --- | --- |
| **aa** | **E-site** | **P-site** | **A-site** | **E-site** | **P-site** | **A-site** |  |
| **A** | 1.27 | 1.16 | 1.48 | 0.66 | 0.37 | 0.59 | |
| **C** | 1.56 | 0.19 | 2.33 | 0.94 | 2.02 | 0.54 | |
| **D** | 0.77 | 0.25 | 1.19 | 0.97 | 1.32 | 0.78 | |
| **E** | 1.17 | 0.97 | 0.21 | 0.71 | 0.24 | 1.44 | |
| **F** | 0.63 | 1.52 | 2.56 | 0.79 | 0.10 | 0.49 | |
| **G** | 1.06 | 0.95 | 0.63 | 1.23 | 1.32 | 2.29 | |
| **H** | 0.75 | 0.63 | 5.40 | 0.57 | 0.26 | 0.16 | |
| **I** | 1.03 | 2.70 | 1.52 | 1.00 | 0.13 | 0.56 | |
| **K** | 1.60 | 0.90 | 0.70 | 0.80 | 0.89 | 0.85 | |
| **L** | 0.82 | 1.71 | 0.33 | 0.94 | 0.16 | 1.01 | |
| **M** | 1.25 | 6.39 | 2.27 | 1.41 | nan | 0.35 | |
| **N** | 0.90 | 0.19 | 2.02 | 0.91 | 2.41 | 0.57 | |
| **P** | 1.11 | 0.21 | 1.05 | 2.35 | 5.48 | 1.74 | |
| **Q** | 0.93 | 0.42 | 0.60 | 0.97 | 0.58 | 0.58 | |
| **R** | 1.12 | 1.02 | 0.31 | 0.99 | 1.25 | 1.39 | |
| **S** | 0.80 | 0.69 | 0.78 | 1.00 | 0.41 | 0.84 | |
| **T** | 1.05 | 0.68 | 0.62 | 0.78 | 0.47 | 0.88 | |
| **V** | 0.95 | 2.39 | 1.09 | 1.43 | 0.12 | 0.70 | |
| **W** | 0.48 | 0.16 | 2.54 | 1.11 | 0.41 | 1.75 | |
| **Y** | 0.36 | 0.66 | 0.84 | 1.10 | 0.82 | 0.93 | |

**Supplementary Table S3. Overrepresentation of amino acids in E-, P-, A-sites upon eIF5A depletion**^23^**.** Overrepresentation is calculated as the ratio of frequencies of a given amino acid in the foreground (FD_P-site codon_ > 2 or FD_P-site codon_ < -2) in relation to the background (0.5 < FD_P-site codon_ > -0.5).

|  | FD_P-site codon_ > 2 | | | FD_P-site codon_ < -2 | | |
| --- | --- | --- | --- | --- | --- | --- |
| **aa** | **E-site** | **P-site** | **A-site** | **E-site** | **P-site** | **A-site** |
| **A** | 0.53 | 0.55 | 0.55 | 0.78 | 1.76 | 1.25 |
| **C** | nan | nan | 0.17 | 2.57 | 6.90 | 2.69 |
| **D** | 1.45 | 2.21 | 0.65 | 0.51 | 0.87 | 0.53 |
| **E** | 0.55 | 0.29 | 0.65 | 0.98 | 2.81 | 0.90 |
| **F** | 0.20 | 0.49 | 0.68 | 1.82 | 0.73 | 1.66 |
| **G** | 0.86 | 1.24 | 1.25 | 0.83 | 0.86 | 0.44 |
| **H** | 0.28 | 0.34 | 0.55 | 1.49 | 1.68 | 1.29 |
| **I** | 0.79 | 0.96 | 2.16 | 1.13 | 0.30 | 0.98 |
| **K** | 0.45 | 0.18 | 0.82 | 2.30 | 5.91 | 3.30 |
| **L** | 0.21 | 0.74 | 1.57 | 1.23 | 0.21 | 1.21 |
| **M** | 0.35 | 0.16 | 0.40 | 0.95 | 1.06 | 2.53 |
| **N** | 0.50 | 1.09 | 0.91 | 1.40 | 0.59 | 0.63 |
| **P** | 7.89 | 4.02 | 2.76 | 0.34 | 0.66 | 0.51 |
| **Q** | 0.13 | 0.34 | 0.74 | 1.49 | 0.92 | 0.17 |
| **R** | 0.51 | 0.28 | 0.89 | 1.55 | 3.46 | 4.53 |
| **S** | 0.48 | 0.48 | 0.34 | 0.52 | 0.87 | 0.63 |
| **T** | 0.34 | 0.27 | 0.69 | 0.62 | 0.43 | 0.30 |
| **V** | 0.95 | 0.67 | 0.84 | 0.88 | 0.47 | 0.85 |
| **W** | 0.26 | 0.84 | 1.27 | 0.85 | 0.73 | 0.65 |
| **Y** | 0.30 | 0.54 | 0.46 | 0.92 | 0.25 | 2.19 |

**Supplementary references**

1 Anand, M., Chakraburtty, K., Marton, M. J., Hinnebusch, A. G. & Kinzy, T. G. Functional interactions between yeast translation eukaryotic elongation factor (eEF) 1A and eEF3. *J Biol Chem* **278**, 6985-6991 (2003).

2 Anand, M., Balar, B., Ulloque, R., Gross, S. R. & Kinzy, T. G. Domain and nucleotide dependence of the interaction between *Saccharomyces cerevisiae* translation elongation factors 3 and 1A. *J Biol Chem* **281**, 32318-32326 (2006).

3 Kotter, P., Weigand, J. E., Meyer, B., Entian, K. D. & Suess, B. A fast and efficient translational control system for conditional expression of yeast genes. *Nucleic Acids Res* **37**, e120 (2009).

4 Johnston, J. A., Johnson, E. S., Waller, P. R. & Varshavsky, A. Methotrexate inhibits proteolysis of dihydrofolate reductase by the N-end rule pathway. *J Biol Chem* **270**, 8172-8178 (1995).

5 Bachmair, A., Finley, D. & Varshavsky, A. *In vivo* half-life of a protein is a function of its amino-terminal residue. *Science* **234**, 179-186 (1986).

6 Sikorski, R. S. & Hieter, P. A system of shuttle vectors and yeast host strains designed for efficient manipulation of DNA in *Saccharomyces cerevisiae*. *Genetics* **122**, 19-27 (1989).

7 Brachmann, C. B. *et al.* Designer deletion strains derived from Saccharomyces cerevisiae S288C: a useful set of strains and plasmids for PCR-mediated gene disruption and other applications. *Yeast* **14**, 115-132 (1998).

8 Christianson, T. W., Sikorski, R. S., Dante, M., Shero, J. H. & Hieter, P. Multifunctional yeast high-copy-number shuttle vectors. *Gene* **110**, 119-122 (1992).

9 Longtine, M. S. *et al.* Additional modules for versatile and economical PCR-based gene deletion and modification in *Saccharomyces cerevisiae*. *Yeast* **14**, 953-961 (1998).

10 Boeke, J. D., LaCroute, F. & Fink, G. R. A positive selection for mutants lacking orotidine-5'-phosphate decarboxylase activity in yeast: 5-fluoro-orotic acid resistance. *Mol Gen Genet* **197**, 345-346 (1984).

11 Malone, B. *et al.* Bayesian prediction of RNA translation from ribosome profiling. *Nucleic Acids Res* **45**, 2960-2972 (2017).

12 Langmead, B. & Salzberg, S. L. Fast gapped-read alignment with Bowtie 2. *Nat Methods* **9**, 357-359 (2012).

13 Pertea, M., Kim, D., Pertea, G. M., Leek, J. T. & Salzberg, S. L. Transcript-level expression analysis of RNA-seq experiments with HISAT, StringTie and Ballgown. *Nat Protoc* **11**, 1650-1667 (2016).

14 Quinlan, A. R. & Hall, I. M. BEDTools: a flexible suite of utilities for comparing genomic features. *Bioinformatics* **26**, 841-842 (2010).

15 Trapnell, C. *et al.* Transcript assembly and quantification by RNA-Seq reveals unannotated transcripts and isoform switching during cell differentiation. *Nat Biotechnol* **28**, 511-515 (2010).

16 Balakrishnan, R. *et al.* YeastMine - an integrated data warehouse for *Saccharomyces cerevisiae* data as a multipurpose tool-kit. *Database (Oxford)* **2012**: bar062, (2012).

17 Jones, E., Oliphant, E. & Peterson, P. *SciPy: Open Source Scientific Tools for Python*, <http://www.scipy.org/> (2001).

18 Robinson, J. T. *et al.* Integrative genomics viewer. *Nat Biotechnol* **29**, 24-26 (2011).

19 Marks, J. *et al.* Context-specific inhibition of translation by ribosomal antibiotics targeting the peptidyl transferase center. *Proc Natl Acad Sci U S A* **113**, 12150-12155 (2016).

20 Kannan, K. *et al.* The general mode of translation inhibition by macrolide antibiotics. *Proc Natl Acad Sci U S A* **111**, 15958-15963 (2014).

21 O'Shea, J. P. *et al.* pLogo: a probabilistic approach to visualizing sequence motifs. *Nat Methods* **10**, 1211-1212 (2013).

22 Schwartz, D. & Gygi, S. P. An iterative statistical approach to the identification of protein phosphorylation motifs from large-scale data sets. *Nat Biotechnol* **23**, 1391-1398 (2005).

23 Schuller, A. P., Wu, C. C., Dever, T. E., Buskirk, A. R. & Green, R. eIF5A Functions Globally in Translation Elongation and Termination. *Mol Cell* **66**, 194-205 e195 (2017).
